# Actuation Mechanism of Microvalves: A Review

**DOI:** 10.3390/mi11020172

**Published:** 2020-02-07

**Authors:** Jin-Yuan Qian, Cong-Wei Hou, Xiao-Juan Li, Zhi-Jiang Jin

**Affiliations:** 1 Institute of Process Equipment, College of Energy Engineering, Zhejiang University, Hangzhou 310027, China; qianjy@zju.edu.cn (J.-Y.Q.); lixiaojuan@zju.edu.cn (X.-J.L.); jzj@zju.edu.cn (Z.-J.J.); 2 State Key Laboratory of Fluid Power and Mechatronic Systems, Zhejiang University, Hangzhou 310027, China

**Keywords:** microvalves, actuation mechanism, material, application

## Abstract

The microvalve is one of the most important components in microfluidics. With decades of development, the microvalve has been widely used in many industries such as life science, chemical engineering, chip, and so forth. This paper presents a comprehensive review of the progress made over the past years about microvalves based on different actuation mechanisms. According to driving sources, plenty of actuation mechanisms are developed and adopted in microvalves, including electricity, magnetism, gas, material and creature, surface acoustic wave, and so on. Although there are currently a variety of microvalves, problems such as leakage, low precision, poor reliability, high energy consumption, and high cost still exist. Problems deserving to be further addressed are suggested, aimed at materials, fabrication methods, controlling performances, flow characteristics, and applications.

## 1. Introduction

As an emerging technology, microfluidic manipulates small amounts of liquid utilizing microchannel with tens to hundreds of microns in size. Microfluidics systems are widely used in many fields, including biomedical engineering, chemical engineering, etc. Liquid-liquid two phase flow in the microchannel is the typical phenomenon in the microfluidic systems. Flow pattern, mass transfer, and mixing efficiency are the most important considerations for two-phase flow [1,2,3,4]. Most microfluidic systems use lithography for manufacturing, and they are characterized by miniaturization, automation, integration, and portability. Compared to traditional experimental equipment, microfluidic systems have obvious advantages: low cost (a little amount of sample), high precision, small space, effective flow control, etc. In order to achieve fluid control and operation at a microscopic scale, there are several main components that constitute the microfluidic systems, such as microsensors, micropumps, microvalves, micromixers, and microchannels.

The microvalve is one of the most important components in microfluidic systems, and its functions include flow regulation, on/off switching, sealing of biomolecules, micro/nano particles, chemical reagents, etc. The main properties of existing microvalves include low leakage, small dead volume, low power consumption, insensitivity to particle contamination, fast response, and linear operation. Based on their structure, microvalves can be divided into two kinds: active microvalves and passive microvalves. The active microvalve needs to control the microfluid with a driving device, and the passive microvalve can generally control the microfluid by the back pressure. In addition, according to the initial state, the microvalve can be divided into two kinds: normally open type and normally closed type.

As for microvalves, the application and the function of them are depended on their structures, and their structures are related to their actuation mechanisms. For example, the micro Tesla valve is comprised of three sections of microchannels. When the fluid flows in the forward direction, the total pressure loss is small, but when the fluid flows in the reverse direction, the loss is great. Thus, the Tesla valve is a typical microvalve without external force and it is applied to the hydrogen fuel cell [5,6,7]. In fact, microvalves have no fixed structure, but almost have the microchannel and the substrate. Most of them also have a membrane to control the opening and closing of the microchannel. Many scholars have studied the application of valves with different actuation mechanisms, mainly focusing on bioengineering and chemical engineering. In the bioengineering field, microvalves are worked as medical devices for the treatment of human diseases based on some special actuation mechanism, and some of them are used in drug delivery applications [8]. As for the patients who suffer from hepatocellular carcinoma (HCC), they had to complete a liver transplant and transarterial chemoembolization (TACE) was needed after the surgery. Titano et al. invented an end-hole versus microvalve infusion catheter to optimize the effectiveness of TACE [9]. Oh et al. presented a 3D dome petal shaped polydimethylsiloxane (PDMS)/Parylene microvalve for hydrocephalus (a pathological condition) [10,11]. Galanopoulos et al. showed an assembled micro check valve using the two-photon polymerization technique, and the valve was implanted in veins of patients whose natural check valves malfunction in the future [12,13]. Chen et al. showed a floating-disk self-regulating parylene microvalve to control the biomedical flow, especially for intraocular pressure (IOP) regulation in glaucoma patients by controlling the eye fluid drainage [14,15]. Moreover, there are also many microvalves for bioassay and sampling equipment. Szydzik et al. reported that the V-gate elastomeric microvalve affected haemocompatibility, and the microvalve based on pneumatic actuation could realize rapid switching control for blood sample delivery [16]. Cheng et al. presented a normally closed plunger microvalve based on electrical self-actuation, for in vivo and point-of-care diagnostic application [17]. Lv et al. showed a cam structured medical digital microvalve, which worked as the core of flow control systems for clinical and surgical transfusion devices [18]. Augustine et al. displayed a low-cost all-plastic microvalve array for multiplexed immunoassay that measure the presence and concentration of multiple harmful agent [19]. Landari et al. presented a unidirectional embedded microvalve which was connected to a miniaturized micropump, for drug delivery and low pressure biomedical applications [20].

In chemical engineering, as an important part of many experimental instruments (like lab-on-a-chip), scholars have proposed a number of microvalves based on specific actuation mechanisms to achieve specific functions. Sung et al. developed a low-cost PDMS microvalve to control multiple reagents for enzyme-linked immunosorbent assay (ELISA) on a programmable lab-on-a-chip (LOC) platform [21]. Flores et al. presented a low consumption single-use microvalve, which was used to impulse small volumes of fluids, and it had been designed to be highly integrable in printed circuit boards (PCB) based microfluidic platforms [22]. Lee et al. proposed a 3D-printed quake-style microvalve, which provided a significant improvement to the performance of equipment such as microscopes and piping simplicity for future large-scale arrays [23]. Wang et al. investigated a new microvalve design of an elastomer normally closed microvalve, which was widely used in high density microfluidics systems to minimize actuation pressures and ensure reliable operation [24]. Li et al. studied a pneumatic microvalve controlled microfluidic chip, which provided precise fluidic control for cell manipulation in divisional cell co-culture [25]. Liu et al. researched a pneumatic control method of a novel electromagnetic microvalve based on microchamber air pressure to improve controlling precision in microfluidic chips [26]. Based on liquid surface tension sealing of the molten solder, Yang et al. proposed a resealable, ultra low-leak microvalve for vacuum applications [27]. Tahvildari et al. demonstrated a microfluidic device which consisted of multiple microvalves and an array of nanopores, and the device could realize the precise manipulation of fluidic and electrical access to various regions of the embedded silicon nitride (Si-N) membrane [28]. Manginell et al. invented a phase-change microvalve for greenhouse gas (GHG) sampling, and the valve had low leakage rate and a long storage time [29].

Due to various kinds and complex structures, there is currently no article to categorize newly emerged microvalves in the past decade based on actuation mechanisms. Thus, this paper mainly focuses on actuation mechanisms of microvalves, and classifies different microvalves by different driving sources, including electricity, gas, magnetism, material and creature, surface acoustic wave (SAW), etc. In addition, the materials required for each actuation mechanism and corresponding applications are also presented in this paper. The review of microvalves is of benefit to help readers understand how microvalves work. This paper also discusses the disadvantages of existing microvalves and proposes the direction for improvement in the future.

## 2. Actuation Mechanisms of Microvalves

According to different driving sources, the actuation mechanisms of microvalves are divided into five aspects in this paper, including electricity, magnetism, gas, material and creature, and surface acoustic wave (SAW). Different applications require different actuation mechanisms. For example, some devices cannot be connected to an external power supply, thus the microvalve may be actuated by magnetism; pH-sensitive actuation and biology actuation are used to meet the requirements of no energy consumption; some experiments are dangerous, a light-actuated microvalve or a SAW microvalve can be utilized in the experiment. In this section, microvalves based on different actuation mechanisms are summarized from application, structures, fabrication methods, materials, advantages, disadvantages and so on. The selection of energy-efficient and efficient actuation mechanisms is an important research direction for future microvalve research.

### 2.1. Electricity Actuation

#### 2.1.1. Electrostatic Actuation

The electrostatic microvalve mainly comprises a valve-closing electrode, a valve-opening electrode, and a flexible movable membrane. The operation of the valve is realized by controlling the voltage, which is applied to the membrane. The equation for the electrostatic force between two electrodes is given by

(1) $$F_{e}=\left(\varepsilon_{0}\varepsilon_{a}AV^{2}\right)/\left(2g^{2}\right)$$

where *ε_o_* is the vacuum permittivity, *ε_a_* is the dielectric constant of the air between electrodes, *A* is the area of the electrode, *V* is the voltage across the electrodes, and *g* is the total distance between the electrodes [30].

A theoretical model was made to predict the closing voltage when the valve is completely open. A force balance was performed on the membrane and can be represented by

(2) $$F_{e}+F_{s}+F_{a}=0$$

There are three forces acting on the valve membrane: the electrostatic force, *F_e_*, pulling up on the membrane, the spring force, *F_s_*, pulling up on the membrane and the force from the air pressure, *F_a_*, pushing down on the membrane [30].

The response time of the valve is short, and the energy consumption of it is low. When the valve is used to control fluid flow, the applied voltage is high. Thus, this type of valve is mainly used to controls high pressure airflow. Some electrostatic microvalves can withstand pressures up to 126 kPa [31]. Electrostatic microvalves are mostly normally-closed microvalves.

**Figure 1:**
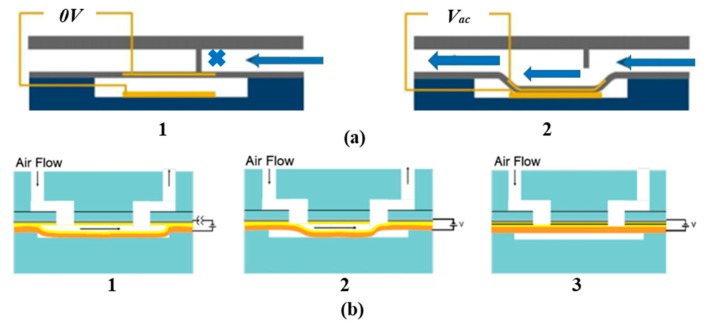
Comparison of the valve operation between normally-closed microvalve (**a**) and normally-opend microvalves (**b**). (**a**) 1 Voltage off, valve fully open; 2 Voltage on, valve starting to close; 3 Voltage on, valve fully closed [30]; (**b**) Operation mode [32].

**Figure 2:**
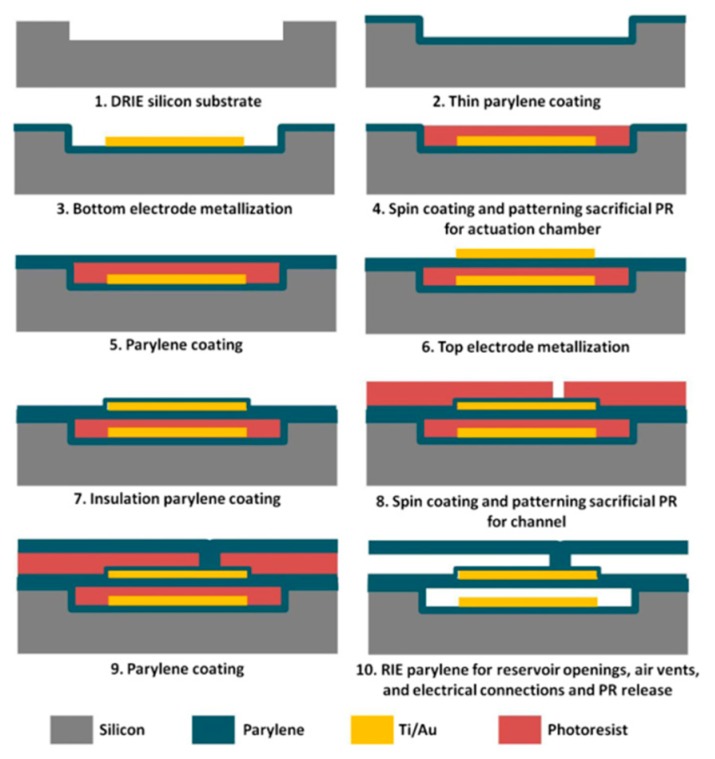
Fabrication process flow of electrostatic actuated microvalve [32].

Structures of the normally-closed electrostatic microvalve and the normally-opened electrostatic microvalve do not have obvious differences from Figure 1. The biggest difference is the original shape of the membrane, where one of the normally-closed microvalve is flat and the one of the normally-opened is concave typed [30,32]. Due to the small size and a large number of components in the microvalve, the fabrication process flow of an electrostatic actuated microvalve is displayed in Figure 2. The installation sequence of the micro valve is from bottom to top, and actuation chamber is filled with a sacrificial photoresist layer (PR) by spinning and patterned. There are many materials used to assemble microvalves, including the silicon substrate, Ti/Au electrodes, and the thin parylene layer. Some special processing technologies are used to process components of microvalves, such as deep reactive ion etched (DRIE), and reactive ion etching (RIE) [32].

Bae et al. introduced a novel bidirectional electrostatic microvalve for controlling high pressure gas. The advantage of this valve was its microsecond switching performance [31]. The experiment showed that the valve can open or close in 50 µs or less for applied pressures up to 126 kPa, and no leakage was found when the pressure is up to 1.1 MPa. The prototype valve had been opened and closed 47,000,000 times before failure. Dankovic et al. developed a thermoplastic normally-opened microvalve using electrostatical operation [33]. The new fabrication method based on Universal Laser System CO_2_ laser was applied to weld thermoplastic films. Unlike usual electrostatic microvalves, the valve did not have the movable membrane and the channel walls collapsed toward each other when the specified voltage is applied. The measurements showed that the leak rate was less than 10% of the flow rate. Messner et al. proposed a normally-closed three-way microvalve based on electrostatic actuation [34]. The fabrication of the valve used silicon micromachining to make the stack of silicon chip install onto a ceramic substrate. Because of numerous advantages, such as the small size and the fast response, the valve was applied to gas controlling application, even the space travel mission. Tice et al. proposed an electrostatic microvalve based on elastomer. The microvalve could resist pressures up to 3 kPa with a voltage of 220 V. Replica molding, plasma bonding, and micro-transfer printing were used to manufacture this valve and it was integrated on a single chip [35]. Yoshida et al. proposed an electrostatic microvalve based on a new pressure balance mechanism and the electrostatic actuator. The valve was applied to adjust the concentration of methanol in direct methanol fuel cell (DMFC) systems [36]. The input pressure of the valve could be up to 40 kPa, and the driving voltage ranged from 50 V to 110 V. Moreover, the novel design could satisfy many industrial requirements, like low energy consumption operation and standard mass production.

#### 2.1.2. Electrochemical Actuation

Electrochemical microvalves are considered as a highly integrated component with very low dead volume and power requirements. Embedding in a microfluidic network can be readily accomplished, owing to its out-of-plane architecture that allows monolithic fabrication of the valve and the surrounding microfluidic network. The valve features a compact actuator, negligible footprint, and mass-production capabilities. These characteristics make the valve particularly suited for lab-on-a-chip applications [37].

**Figure 3:**
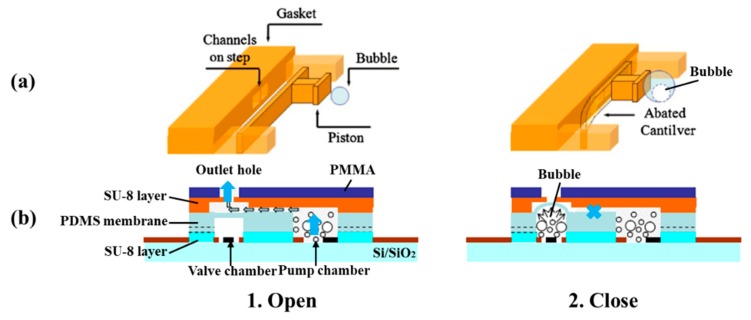
Two types of schematic diagrams of electrochemical actuated microvalves. (**a**) A microvalve based on electrochemically actuated SU8 cantilever [37]; (**b**) A microfluidic valve based on electrochemical (ECM) actuated membrane [39].

Unlike electrostatic microvalves, electrochemical microvalves use electrodes to electrolyze solution, such as NaCl solution, to produce hydrogen bubbles [38]. Figure 3 shows two types of schematic diagrams of the electrochemical actuated microvalve: one is the microvalve based on electrochemically actuated SU8 cantilevers [37], the other is a microfluidic valve based on electrochemical (ECM) actuated membrane [39]. The gas is produced by electrolysis to make the membrane deflect in the electrochemical microvalve. The microvalve consists of an electrochemical (ECM) actuator, a flexible polydimethylsiloxane (PDMS) membrane (or a SU8 cantilever) and a micro chamber. The actuator has a chamber containing a redox couple in solution, and the volume of the solution is defined by the required actuation volume of the valve cycle [37]. The ECM actuator used for the valve operation works in potentiostatic mode. A constant voltage is applied across the working and reference electrodes while current is manipulated. The ECM actuation is based on the reproducible production and consumption of hydrogen bubbles at the working and auxiliary Pt electrodes because of reduction or oxidation. The redox reactions can be represented by:

(3) $$O^{n+}+ne^{-}↔R_{ed}$$

where *O^n+^* is the oxidized and R_ed_ is the reduced form of the redox couple, and *n* is the number of electrons involved in the reaction. The equilibrium potential is given by the Nernst equation:

(4) $$E_{e}=E^{0}+\frac{RT}{NF}\cdot \ln\frac{C_{o}}{C_{R}}$$

where *E*^0^ is the standard redox potential; *N* is the mole number; *F* is Faraday’s constant; *R* is the universal gas constant; *T* is temperature of the solution; and *C_o_* and *C_R_* are the concentrations of oxidized and reduced components, respectively [39]. The electrochemical nature of the actuator allows for precise control of the valve diaphragm by controlling the actuation voltage. Actuation is achieved when the system is perturbed from the equilibrium potential resulting in either bubble formation or consumption.

A low power, low dead volume electrochemical microvalve could realize precise control of diaphragm motion by changing actuation voltage [38]. The prototype one could be driven by AAAA battery, and it resisted high backpressure (5 psi) and could be operated for a long time (10 h). The valve was suitable for mass production by molding, and was applied to a small size drug delivery device. Ezkerra et al. introduced a microvalve with the electrochemical actuated SU8 cantilever for lab-on-a-chip applications [37]. The main function part was an actuator with two electrodes, which produced bubbles to push the cantilever for preventing the flow of fluid. Effective sealing with negligible leakage could be achieved up to 20 kPa in this valve. Lee et al. showed an electrochemical microvalve which was manufactured by UV-LIGA microfabrication technologies [39]. The nano particles coated on the working electrode were benefit for faster reversible electrolysis and faster valve operation. From experiment results, 300 µm deflection of valve membrane was achieved when the bias voltage was −1.5 V, and the operation speed of this valve should be improved in the future.

#### 2.1.3. Piezoelectric Actuation

**Figure 4:**
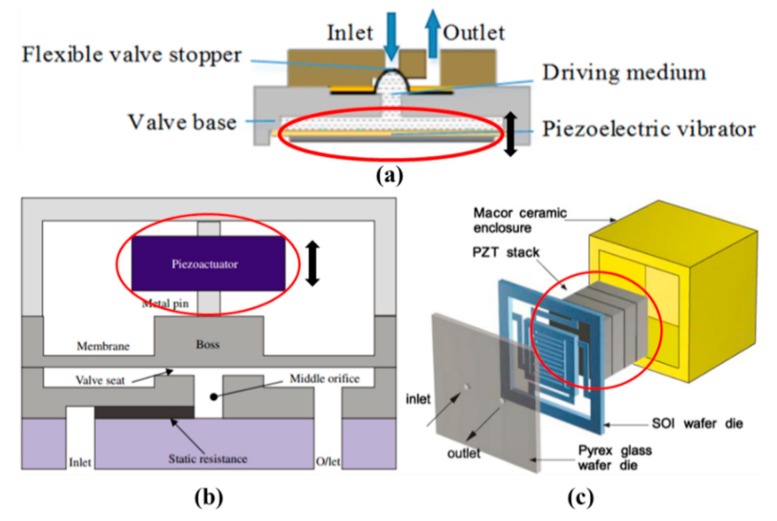
Typical structures of piezoelectric microvalves: (**a**) a normally-closed piezoelectric microvalve with flexible stopper [40]; (**b**) a high pressure piezoelectric actuated microvalve [41]; (**c**) A piezoelectric microvalve for cryogenic applications [42].

**Figure 5:**
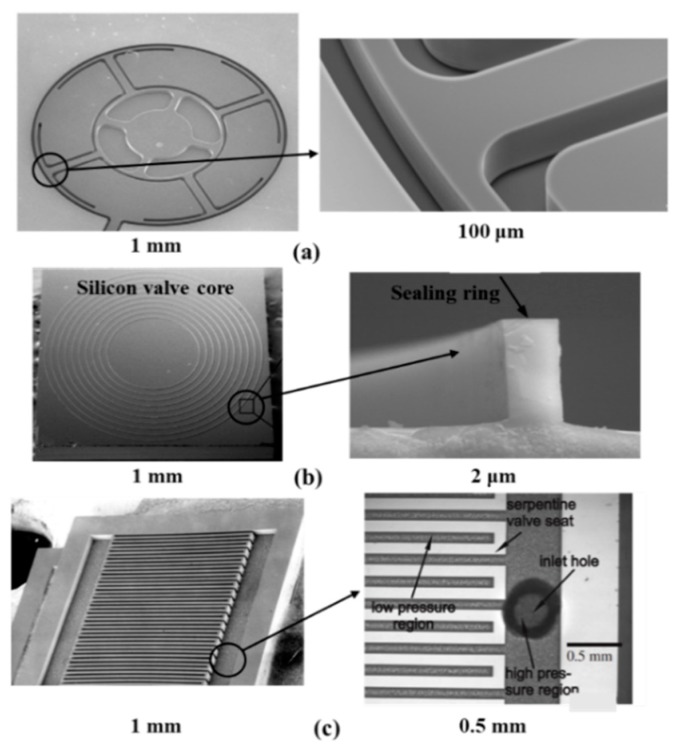
SEM photographs of components in piezoelectric microvalve: (**a**) the microchannel [43]; (**b**) the silicon valve core and the sealing ring [44]; (**c**) the hole with serpentine groove patterns [42].

When piezoelectric actuation is utilized in microvalves, it can produce big bending force (several MPa) and small displacements. The response time of it is relatively small. The crystal inside the valve can produce mechanical stress or stretching with an applied electric field. The voltage is large, but the membrane only takes the place of a small deviation. Although large force is available using piezoelectric actuators, a large stroke is a challenging issue even for large voltages, and the shortcomings of small strokes have been overcome by the hydraulic amplification of stacked piezoelectric discs and piezo bimorphs. There are many different structures of piezoelectric microvalves, which are displayed in Figure 4. They all consisted of the piezoactuator (the piezoelectric vibrator) [40], the valve membrane (the valve plate or flexible valve stopper) and valve seat [41]. Further, SEM photographs of components in piezoelectric microvalves are shown in Figure 5. The most important component in the piezoelectric microvalve is the piezoactuator, which can be called the PZT stack [42]. Specifications of a typical piezoactuator are listed in Table 1.

Take the normally-closed piezoelectric microvalve with flexible stopper as an example, the mechanical model of the piezoelectric microvalve is built to understand the working principle of it [40]. The drive force and the vibration of valve stopper can be expressed:

(5) $$F\left(t\right)=F_{0}\sin\omega t=m\ddot{X}+c\dot{X}+KX$$

where *F*(*t*) is the force of mass block, *F*_0_ is maximum output force of piezoelectric vibrator, *m* is the equivalent mass of flexible valve plug and additional fluid, *K* is the equivalent rigidity of the flexible valve stopper, *c* is the equivalent damping modulus which is produced by the interaction between the flexible valve stopper and the fluid.

Setting the steady state response of valve stopper is:

(6) $$X_{p}(t)=A_{v}\sin(\omega t-\phi )$$

where *A_v_* is the amplitude of valve stopper and Φ is phase shift, which are expressed as

(7) $$A_{v}(\omega )=\frac{F_{0}}{m}\frac{1}{\sqrt{{\left[1-\left(\frac{\omega }{\omega_{n}}\right)\right]}^{2}+{\left[2\zeta \left(\frac{\omega }{\omega_{n}}\right)\right]}^{2}}}$$

(8) $$\phi \left(\omega \right)=\arctan\frac{2\zeta \left(\frac{\omega }{\omega_{n}}\right)}{1-{\left(\frac{\omega }{\omega_{n}}\right)}^{2}}$$

(9) $$F_{0}=U_{0}G$$

(10) $$\omega_{n}=\sqrt{K/m}$$

(11) $$\zeta =c/2\sqrt{m/K}$$

where *ω_n_* is natural frequency with no damping, *ζ* is the damping factor, *U*_0_ is the amplitude of driving voltage, and *G* is the constant which is related to the material and structure of the piezoelectric vibrator.

When the driving frequency is constant, the response amplitude of the valve stopper *A_v_* increases with *U*_0_ increasing. Because the driving frequency of the piezoelectric vibrator is 0–250 Hz, it is much smaller than the fundamental frequency of the piezoelectric vibrator, so the amplitude of the piezoelectric vibrator can be expressed as:

(12) $$A_{p}=U_{0}H$$

where *A_p_* is amplitude of piezoelectric vibrator and *H* is the constant which is related to the material and installation method of the piezoelectric vibrator. Combined, Equations (7), (9) and (12), the hydraulic amplification ratio can be expressed as

(13) $$R_{A}=\frac{A_{v}\left(\omega \right)}{A_{p}}=\frac{G}{Hm}\frac{1}{\sqrt{{\left[1-\left(\frac{\omega }{\omega_{n}}\right)\right]}^{2}+{\left[2\zeta \left(\frac{\omega }{\omega_{n}}\right)\right]}^{2}}}$$

From Equation (13), when the micro-valve is driven by sine wave voltage, the amplification ratio *R_A_* will increase with the decrease of the gap between *ω* and *ω_n_*. Further, *R_A_* reaches the maximum when the driving frequency *ω* is the same as the nature frequency *ω_n_* [40].

**Figure 6:**
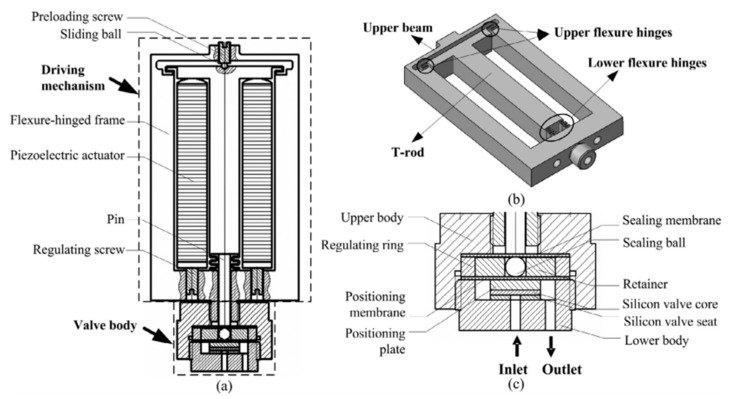
Schematic structure of the microvalve [44]: (**a**) The structure of the microvalve; (**b**) The diagram of the 3D geometry model of the frame; (**c**) The cross-sectional view of the valve body.

The dispensing behavior of the piezo-actuated micro dispensing valve was researched by simulation and experiments [45]. The purpose of research was to make sure that the valve had the large working range, and kept a high precision. The results revealed that when the opening time was shorter than 10 ms, the transient behavior after valve opening strongly effected the time. The group of Fazal developed a novel normally open piezoelectric microvalve based on the concepts of micro and fine machining [46,47]. The design of valve realized a wide control range of high-pressure gas flow, when the pressure difference between inlet and outlet was high. The advantages of this valve included low power consumption, continuous control and precise control. Groen et al. presented a new type of piezo-actuated microvalve combining with capacitive displacement sensing [43]. The main manufacturing methods of it were one release etch and deep reactive ion etching (DRIE). The device was used to monitor arterial blood pressure waveform. Various microvalves have been developed to control the flow rate of propellants in the micro-satellites, and Lv and Zhang proposed a piezoelectric microvalve based on a microfabricated silicon valve seat. As shown in Figure 6, the silicon seat was deposited with parylene, so the valve seat could meet the sealing requirement after 10^5^ cycle operations and improve its fatigue performance. A flexure-hinged frame and the piezoelectric actuator could make sure that the valve realized rapid adjustment. A silicon sealing pair guaranteed the low degree of leakage and a small geometrical deformation in the valve [44,47]. Yang et al. studied a novel piezoelectric microvalve with many tight seating rings to ensure the low leakage. The valve was mainly used to proportionally control liquid flow, and the model was simulated by CFD to predict the flow field. This valve technology was likely to utilize in the precise control for large spaceship [48].

As for a distributed cooling system in cryogenic applications, the group of Park proposed a piezoelectric microvalve with embedded sensors for flow control. The valve comprised a piezoelectric stack actuator, the ceramic encapsulation, a silicon-on-insulator wafer and a glass wafer. When the operation temperature was 80–380 K, the valve could function normally as designed, and sensors also worked well in this range. Moreover, they also studied the performances of different types of gases using the designed valve, in conditions of low and room temperature. The experiment data agreed well with the result of simulation [42,49,50,51]. Rakotondrabe et al. presented a novel piezoelectric microvalve combining with an unimorph bending cantilever. The function of this device was smooth and rapid control for fluid flow. Based on some simplifying hypothesis like ideal fluid and so on, the physical model of the valve was established to research dynamic behavior of it [52]. Ramanamurthy et al. reported a normally-closed piezoelectric microvalve fabricated by surface micromachining, molding and diaphragm transfer (AMANDA) process. A mechanical clamp was used to link unimorph and actuator for ensuring the dependability of the valve [53]. Scheuenpflug et al. developed a diaphragm microvalve based on piezo-actuation, which was manufactured by a rapid prototyping method. Whatever the media was, liquid or gas, the valve had a high flowing rate and a low degree of leakage. As a passive module, the valve could be integrated with a microfluldic system, like the production of radiopharmaceutical drugs [54]. Wiederkehr et al. introduced a microvalve based on piezoelectric poly(vinylidene fluoride) (PVDF) to control the gas flow. The device consisted of a glass micronozzle and a piezoelectric actuator formed by two PVDF sheets. When DC voltage was applied to the electrodes of actuator, the whole device could be adjusted, and it was utilized in the application of precise control [55]. Wu et al. studied a piezoelectrical polymer microvalve, which can realize hydraulic amplification. The main innovative points in this valve were that choosing an incompressible elastomer as the medium was the most important thing to change from tiny axial displacement to obvious valve head stroke, and the axial structure of the valve made it possible to form valve arrays [56].

Comparisons of three types of electrically actuated microvalves are shown in Table 2. The high voltage is applied to the microvalve related with electricity. The response times of these microvalves are mostly short. Because the power of these microvalves comes from electricity, it is convenient to operate these valves. Electrical microvalves have a lot of advantages, including satisfactory particle tolerance and low cost, so the valves are widely applied to many fields, like lab-on-a-chip applications, microfluidic devices, direct methanol fuel cell (DMFC) systems, drug delivery system, and even micro-satellites.

### 2.2. Magnetism Actuation

#### 2.2.1. Magnetic Actuation

**Figure 7:**
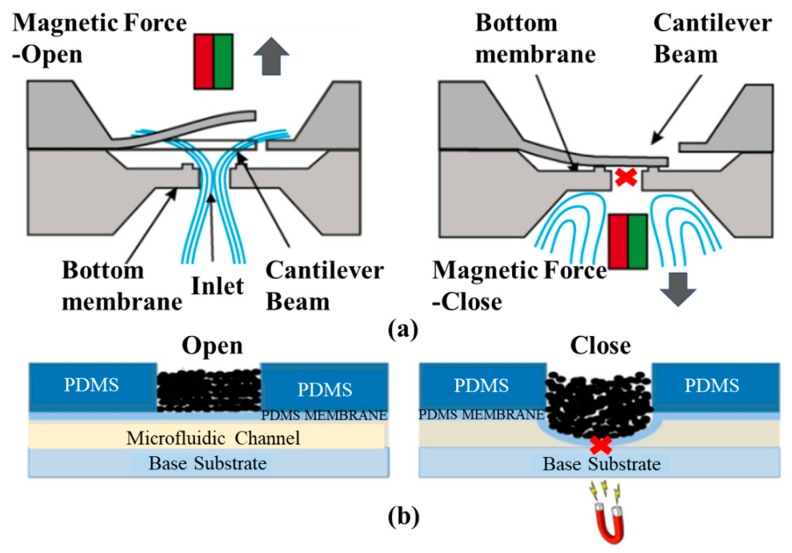
Schematics of two magnetic microvalves: (**a**) an externally magnetic ON/OFF microvalve [57]; (**b**) a magnetic microvalve based on MR fluid [58].

A typical magnetic microvalve contains a permanent magnet and the flexible elastic membrane with the soft magnetic material. The deflection of the membrane is caused by the magnetic forces. Thus, this kind of microvalve only needs less external energy consumption, and they all belong to externally actuated microvalves. To increase magnetic force in the valve, the movable membrane can be integrated with coils. Two different structures of magnetic microvalves are displayed in Figure 7, which showed that the working principle of the magnetic microvalve is simple. Magnetic cantilever beam [57] and magnetic bead [58] are utilized to control microchannel (on/off switching) in the valve, and magnetorheological (MR) fluids micropatterned on top of a PDMS membrane controls the deformation of the membrane under the action of the magnet [59]. Different locations of the magnet define the deflection direction of the membrane and the deflection of the membrane caused by the magnetic force opens or closes the flow channel in the valve. Magnetic actuation simplifies the structure design and reduces components. The operation could be controlled remotely by magnetic fields in some magnetic microvalves. This ensures the safety of operators for some operation of dangerous substances. Due to the fact that the flow channel cannot be completely closed under the action of magnetic force, the biggest shortcoming of this microvalve is the leakage.

**Figure 8:**
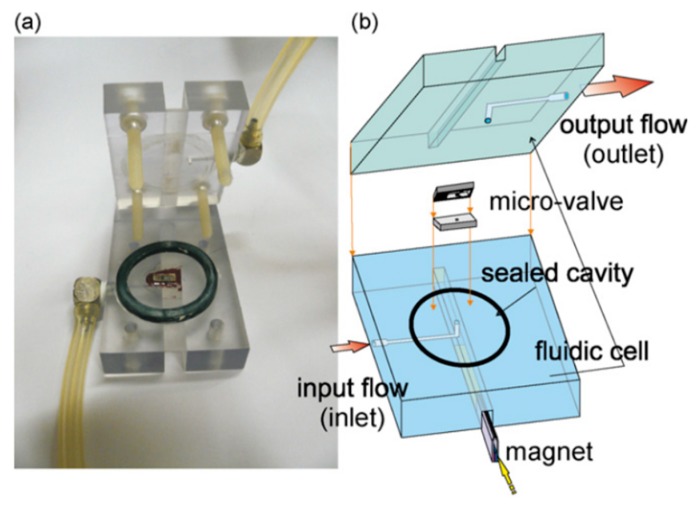
Parts of the magnetic-actuated microvalve and polycarbonate fluidic cell [57]: (**a**) Experimental set-up; (**b**) Schematics of the system.

Many scientists propose different magnetic materials for magnetic microvalves to adapt the different application conditions. As common magnetic materials, Fe, Co, and Ni are widely used in the magnetic microvalve. Other paramagnetic materials are sometimes also added in the core component of the valve. The group of Casals-Terre analyzed an electrodeposited layer of Co-Ni on a V-shaped cantilever beam, which was used in a magnetic microvalve based on a permanent magnet [57,60]. The valve could be used as a check-valve to control the N_2_ flow at the flow rate of 20 sccm. In Figure 8, the experimental set-up of this microvalve showed that the gas flow direction was from the bottom to the top.

Using a paramagnetic bead etched into a substrate of silicon, Chang et al. presented a novel magnetic microvalve. The principle of the whole device was that beads under the valve were magnetized by a permanent magnet to realize bidirectional actuation and decreased required current. Low energy consumption and the simple scalable structure are main advantages of the valve [59]. Okazaki et al. investigated a micro-gas valve adopting a magnetostrictive actuator comprised Fe-Pd and Fe-Ga alloys. By applying a magnetic field parallel to length, the cantilever-type actuator was bent. The valve changed the gas flow rate by adjusting magnetic field strength and the actuator was suitable for application in microfluidic devices [61]. Viard et al. introduced a MEMS magnetic microvalve consisting of a magnetostatic actuator and a packaging, allowing for accurate positioning. The valve was used to provide pulsed jets whose frequency range could change from 0 to 500 Hz, and the velocity of flow could reach 150 m/s. The device satisfied the requirements of aeronautic flow control tests [62].

As for membranes, they would be magnetized or added magnetic materials for operation in the magnetic field. Most membranes were PDMS membranes, and a magnetic microvalve utilizing iron-powder filled PDMS was proposed by Cheng et al. The valve was adjusted by transcutaneous control, and did not need any additional power source, just a magnet. The device was mainly applied to implantable drug delivery system, and a pressurized balloon reservoir was used to pump the drug [63]. According to Gholizadeh et al., the elastomeric membranes of the magnetic microfluidic valve based on magnetorheological fluids did not need to be reprocessed [58]. Polymer microfabrication technologies were used to fabricate the microvalve, which had plenty of advantages, such as simple fabrication, small size, and no power source. The device was suitable for the portable analytical equipment.

#### 2.2.2. Electromagnetic Actuation

The difference between the magnetic actuation and the electromagnetic actuation is the source of the magnetic field. One uses magnetic field and the other uses electromagnetic field. Compared with the magnetic field from the magnet, the electromagnetic field needs external electric energy and strength of it can be controlled by the current intensity, causing the operation of the valve can be more precise. Electromagnetic valve is also an industrial device with electromagnetic control, which is the basic element of automation used to control fluids. Through controlling the switching (on/off) of the electromagnets, the direction of flow could be adjusted. The differences between the two types of magnetic actuated microvalves are shown in Table 3.

Many precise industrial devices need electromagnetic microvalves, like pneumatic pressure control in the lab-on-a-chip [64]. The advantages of this kind of microvalve, including rapid response and high precise control, satisfy most of industry requirements. The core component of some electromagnetic microvalves is the plunger electro-magnet [65]. FLUENT software with the function of UDF was used to simulate the flow field under the function of electromagnetic fields. Wu et al. researched a novel microvalve based on four electromagnets and magnetic fluid, and simulation results showed that performances of 40 mT magnetic field were the best [66]. Burke et al. developed an in-channel magnetic microfluidic system including a microvalve. Combining the permanent magnet with micro scale coils produced a relatively huge magnetic field force, and it placed the magnetic actuator in the fluid channel [67].

**Figure 9:**
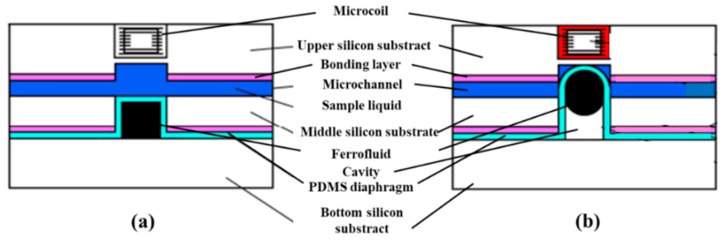
Structure and working principle of microvalve [68]: (**a**) Open state; (**b**) Close state.

There is a special electromagnetic microvalve, which is based on the ferrofluid. Ferrofluids are magnetic liquids created by suspending ferromagnetic particles of 10 nm in a carrier fluid. Carrier fluids can be water, diesters, hydrocarbons or fluorocarbons and favor many different applications. Ferrofluids conform to the channel shape, potentially providing very good seals, and respond to external localized magnetic forces, providing easy actuation. The structure and the working principle of an electromagnetically actuated microvalve based on ferrofluid is shown in Figure 9 [68]. Ferrofluid controlled the deflection of the membrane to adjust fluid flow.

Furthermore, the group of Luharuka presented a bistable electromagnetically actuated rotary gate microvalve based on a suspended gate to adjust flow. An in-plane rotary bistable micromechanism (IPRBM) was used in the gate to constrain its degrees of freedom. The outer electromagnetic actuator was applied to control the valves. Utilizing a Polytec Laser Doppler Vibrometer (LDV) system could acquire the vertical displacement of membrane [69,70]. Kawakami et al. developed a novel electromagnetic actuated sliding valve, which had performances of resisting alkali and acid [71]. Materials and coatings with resistance to acid and alkali were utilized in the valve, and the valve was applied to a “micro beaker process” for realizing even reactions.

### 2.3. Gas Actuation

#### 2.3.1. Pneumatic Actuation

As an important type of microvalves, pneumatic microvalves are widely worked as key components for automating liquid manipulation and flow control in microfluidics. In comparison with other microvalves, this needs an external system, which contains a vacuum pump and a pneumatic actuator. Pneumatic actuators have been applied to various robotic systems, owing to their relatively high power-to-weight ratios [72]. The membrane is also one of the most critical parts of the pneumatic microvalve, such as PDMS layer, silicon membrane, and silicone rubber sheets. These flexible membranes can be deformed by pneumatic actuation, for closing or opening the fluidic channel of the corresponding valve. The operation of the pneumatic microchannel actuator makes the thin membrane bend, resulting in the bending of the liquid microchannel and its closure. Due to inadequate actuation pressure or a thick membrane, the response of the valve would become slow. Undue actuation pressure will make the restoring time of the membrane longer [73]. The membrane thickness, actuation pressure, the configuration, the level of structural complexity and the position of the microvalve in the device influence the dynamics of microvalves. Pneumatic valves based on the compressed air can also be used industrially to control the flow of various types of fluids such as air, water, steam, various corrosive media, muds, oils, liquid metals and radioactive media. An overview of design principles of pneumatic microvalves is given in Table 4 specifying opening and closing pressures [74]. A simple design is given when two crossing channels of which one is pressurized to close the other (No.3) are implemented. For full sealing, rounded fluidic channels are required, which is challenging in microproduction technology. To avoid the need for rounded channels, more complicated valves have been designed, but they require additional material layers (No.2). Improved designs are published with reduced complexity and number of layers (No.1).

**Figure 10:**
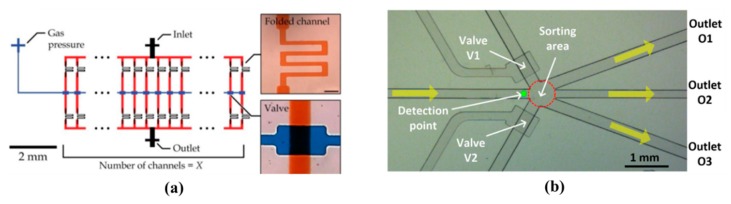
Applications of multiple pneumatic microvalves: (**a**) Integrated microfluidic device containing 100 microvalves [73]; (**b**) Micro-droplet sorter with two pneumatic microvalves [80].

Because of the simple structure and low cost, the pneumatic microvalve is applied to many applications including microfluidic circuits design [75], fuel cell systems [78], mix and sort droplets [79,80], rapid hydrodynamic sample injection [81] and so on. What is more, due to the ease of fabrication and robust operation, microfluidic systems have been developed with the multiple pneumatic microvalves to improve throughput and expand applications [73,80], which are shown in Figure 10.

A microfluidic platform including a pneumatic microvalve could complete electrokinetic sample preconcentration and rapid hydrodynamic sample injection [81]. The valve was fabricated by multilayer soft lithography method to work as a nanochannel preconcentrator, which was used to make the current pass through and hold back flow. This method enabled both rapid analyte concentration and controlled injection volume for high sensitivity. A small-sized pneumatic valve was applied to drive actuator in the wearable robotic system [72]. The most important advantage of valve was light, and the application of it could make the system perform better. For patients whose eyes were impaired, Schneider et al. designed a new grayscale pneumatic microvalve for a reconfigurable tactile tablet. The combination between device and voltage could make tablet generate the same grayscale images [82]. Perdigones et al. reported a pneumatic positive gain microvalve based on PCB substrate, SU-8 and gold. Through experiments, it proved that the device could work as n-channel metal-oxide semiconductor (NMOS). The valve was appropriate to be applied to the fluidics circuits for adjusting fluid flow and microfluidic circuits design [75]. To realize the operation of leak proof and lower pressure damage, Satoh et al. developed a novel microvalve for controlling liquefied gas, whose pneumatic actuation was controlled by two electrostatic sub-valves [78].

In microfluidic systems, the pneumatic microvalve not only is used to merge droplets, but also is applied to sort droplets. The device plays an important role in chemical and biological applications. The device has numerous advantages like high precision and flexible manipulation. A pneumatic horizontal PDMS microvalve was proposed for the droplet merging system [79]. Changing flow resistances of main and side channels could adjust the number and diameter of droplets. The device solved the desynchronization problems, and it could be applied to efficiently mix the droplets in various diameters and numbers without changing the structure of the merging chamber. Chen et al. investigated a sorting droplets microfluidic system based on the bilayer pneumatic microvalve. Because light absorbance of every type droplet was different, changing intensity of light was applied to transfer the droplet to different outlet channels. The current microfluidic systems only needed some modification of structure for achieving droplet sorting function by this sorting method [80].

**Figure 11:**
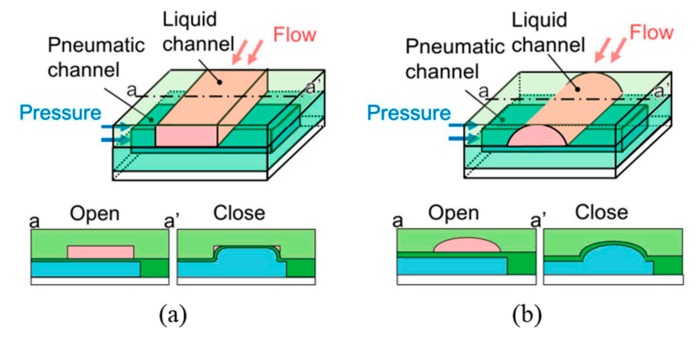
Cross sections of microchannels in pneumatic pressure-driven microvalves [84]: (**a**) Rectangular cross section; (**b**) Semicircular cross section.

Many pneumatic microvalves have multiple-layer structures to adapt to complex conditions. Different manufacture method and materials are used to fabricate the devices. A pneumatically controllable PDMS-based microvalve was utilized to regulate switching of flow using the thick centered membrane, and the valve consisted of the pneumatic layer, the membrane layer, the hole layer and the bas-relief plate [76]. Huang et al. described a novel technology to manufacture a pneumatic microvalve based on the four-layer structure. The connection between the PDMS membrane and the rigid substrate of PMMA ensured the bonding strength to endure high pressure conditions, and the sealing between the control half and the fluid half was reversible [83]. The design not only obviously reduced the time of analysis, but also cut down the cost. A typical three-layer structure pneumatic microvalve was manufactured using inclined lithography method [84]. As shown in Figure 11, the valve comprised a liquid microchannel layer, a thin PDMS membrane layer and a pneumatic microchannel layer (actuator). The liquid channel had a parallelogram-shaped cross section with 500 µm width and 100 µm height. The device was used to convey large cells, such as HeLa cells. Through observing the suspension of the flow of the HeLa cell, it demonstrated the closure of the liquid microchannel. A novel push-down pneumatic microvalve was developed by Park et al., which was is useful in electrochemical microfluidic devices. The bonding material was a mixture of PDMS and hexane. The prototypical device based on proposed fabrication process was proved that it had better performance of valve operation [85].

The pneumatic microvalve also has some shortcomings, such as gas penetration through PDMS membrane. There are two methods avoiding intrusion of gas into the microfluidic channels through PDMS membrane [74]. The first one used an oil droplet, which was placed in the dead end of the pneumatic channel, and it made the microvalve have better sealing capabilities and suppressed the permeability of the valve completely. The second one was based on a parylene coating to make PDMS impermeable to gases. Samuel et al. reported a manufacture method about pneumatic actuated microvalve arrays based on PDMS. Utilizing laser cut molds, the valve could be fabricated easily and rapidly. The method was also introduced to the device which was used to manipulate *C. elegans* [77]. Singh et al. presented a helical pneumatic solenoid micro-valve, which was used to analyze body flow behavior. Through experiments and numerical simulations, when the flow rate was 0.01 mL/h, the valve could block the flow [86].

#### 2.3.2. Thermopneumatic Actuation

**Figure 12:**
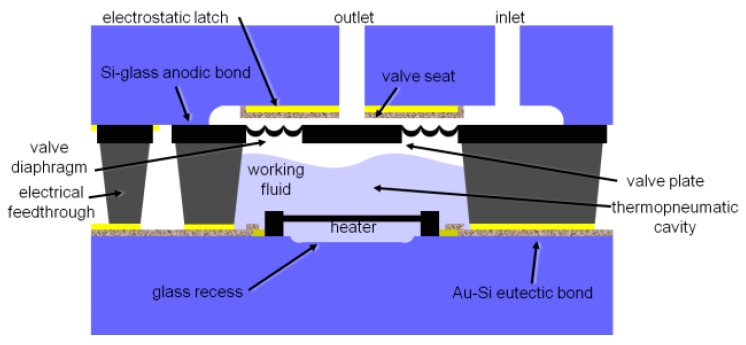
Cross-section view of thermopneumatic microvalve [91].

Unlike the pneumatic microvalves, the key component of the thermopneumatic microvalve is a microheater. As shown in Table 5, thermopneumatic microvalves are widely used in many fields, such as portable SU-8 microfluidic platforms [87], liquid flow control [88], microfluidic chip [89] and so on. A thermopneumatic microvalve consisted of inlet and outlet, an actuation diaphragm, a thermopneumatic actuation chamber, and a thin film heater. In microchannel, fluid is blocked or passed by the motion of actuation diaphragm. Actuation diaphragm is bent up and down by exploiting air expansion that is induced by increasing heater temperature. Figure 12 shows the structure of the thermopneumatic microvalve. Many thermopneumatic actuators are thermoelectrically driven. The phase change liquid could replace the air in the thermopneumatic actuation chamber [90].

Mongpraneet et al. researched a thermopneumatic microvalve consisted of multi-stack PDMS. Fluid flow was controlled through the membrane motion, which was depended on air expansion with heater temperature rising [89]. Many low-cost fabrication processes were applied to this microvalve, like PDMS spinning, oxygen plasma bonding, electroplated micromasking, and thermal evaporation. Perdigones et al. proposed a novel microvalve based on thermo-pneumatic actuation for portable SU-8 microfluidic platforms. The device included two parts, one was a thin SU-8 wall with a gold wire, another one was a pressurized chamber of SU-8. The main advantages of this type valve were getting rid of pressure sources for actuation from outside, low energy consumption and high integrability [87]. Based on the valve plate position sensing and the electrostatic control, a novel thermopneumatic microvalve was introduced by Potkay et al. The small size of the valve (7.5 × 10.3 × 1.5 mm) and low energy consumption was its main advantage [91].

Some bistable microvalves adopting thermopneumatical actuation were also proposed. The bistable microvalve means that the state of the valve is only opening or closing. A drawback of typical active microvalves is that continuous power has to be applied to keep the microvalves open in normally closed microvalves or closed in normally open microvalves. This problem can be solved by bistable actuations that require power only in a transient mode between two stable positions. Thermal buckling of membranes is widely utilized in bistable microvalves. Yang et al. researched a bistable microvalve adopting thermopneumatical actuation, utilizing the fabrication method of sputtering and photolithography [92]. The bistable switching condition was realized through a moving soft magnet and two permanent magnets. The results showed that the heating membrane thickness effected switching time a lot, and no leakage was observed up to a differential pressure of 350 kPa. Based on a thermoelectrically actuated thermopneumatic actuator, another bistable microvalve was manufactured by silicon technology [88]. When back pressure was 150 kPa, the leakage rate would be lower than 1 µL/min. Controlling the valve only needed fairly low energy cost.

### 2.4. Material and Biology Properties Actuation

In this section, microvalves with non-mechanical moving parts will be discussed. Due to some special properties, many materials and creatures are used to actuate microvalves. Actuation mechanisms of phase change materials are discussed, including polymer (hydrogel [93,94,95,96], sol-gel [97]), paraffin [98,99,100,101,102,103], alloy (low melting point alloy [104,105,106] and shape memory alloy [107,108,109,110,111,112]). Compared with the traditional mechanically active microvalves, these phase change microvalves are relatively new and cheap. As a new type of microvalve, the working principle of the bio-actuated microvalve is described in detail [113,114]. Because of their simple device structure, disposability, and low power consumption, these non-mechanical active microvalves are well suited for applications in drug delivery systems [115,116]. Table 6 shows a comparison of microvalves based on material and biology properties.

Stimuli-responsive hydrogel (gel) is able to change its volume reversibly and reproducibly by more than one order of magnitude even with very small alterations of certain environmental parameters. The volume change of hydrogels can be induced in response to a variety of inputs, such as pH, glucose and light. In the following part, mocrovalves based on hydrogel (gel) are discussed in these three stimulus methods.

As a typical phase change material, paraffin can be used either as a propellant for a membrane or as a meltable plug [98,99,100,101,102,103]. Since the volume expansion associated with the solid-to-liquid phase transition of paraffin is 10%–30%, the propellant scheme can be incorporated for the deflection of the membrane.

Metal materials are also widely used to actuate microvalves. For example, the hydrophilia of TiO_2_ is utilized in the light actuated microvalves by irradiating ultraviolet (UV) [117,118,119]. The shape memory effect of shape memory alloy is an attractive actuation principle for the development of microvalves, since it allows simple and compact structures with high output forces, which are capable of controlling high pressure differences and flows [107,108,109,110,111,112]. Due to the high sensitivity of temperature, low melting point alloy phase changes with the change of temperature [104,105,106]. Its advantages, including short manufacturing cycle and low manufacturing cost, make it an important microvalve driving material.

#### 2.4.1. Light Actuation

The microvalves based on light actuation are also referred to as photoresponsive microvalves [94]. The light sources are divided into visible light and invisible light. The invisible light sources include ultraviolet ray and infrared ray. The photoresponsive microvalves are comprised of the light source and the ionic polymer. A quartz halogen illuminator with tungsten filament can be chosen as the light source. The working principle of the microvalve is utilizing the expansion and contraction of the ionic polymer controlled by a single light source. As an externally controlled type of microvalve, photoresponsive microvalves have many advantages that other valves do not have. The optically triggered microvalve permits flexible and remote fluidic handling, and the light actuation did not need physical contact. Because the light source can be installed outside of the valve, it reduces the complexity of the device and the need for integration. The disadvantages of the valve are also evidence of this. Comparing with other microvalves, its opening response time, which is more than 1 s, is relatively long. The closing response time is longer than the opening response time.

Many photoresponsive microvalves use different ionic polymers to improve the performances of the valves. The group of Al-Aribe introduced a hydrogel microvalve activated a porous photoelectric film. The film was used to control the expansion and contraction of a pH sensitive HEMA-AA hydrogel actuator. The self-assembled monolayer of oriented bacteriorhodopsin (bR) purple membrane (PM) patches were immobilized on a porous bio-functionalized gold (Au) surface. Upon irradiation, each bR molecule worked as a proton pump to transports hydrogen ions through a transmembrane ion channel. The results revealed that an 8 µm gap of microchannel could be closed by the valve under a focused light beam [93,94]. Benito-Lopez et al. showed four types of ionogels (ionic liquid polymer gels) applied to light actuated valves. The variation of composition of the ionogels influenced the opening time of the microvalve. The experiment results revealed that the recovery (expansion) process to re-close the channel needed a few minutes, so the microvalve was suited for single-actuation events [95]. Chen et al. reported a light actuated microvalve bearing high leakage pressure. The microvalve based on poly(N-isopropylacrylamide) (PNIPAM) functioned well in cyclic olefin copolymer (COC) microchip. As the thermo-responsive polymer, the pressure-tolerance of PNIPAM could be tuned with the amount of monomer and crosslinker. The microchips with valve were very practical in chemical analysis and proteomic analysis [96].

**Figure 13:**
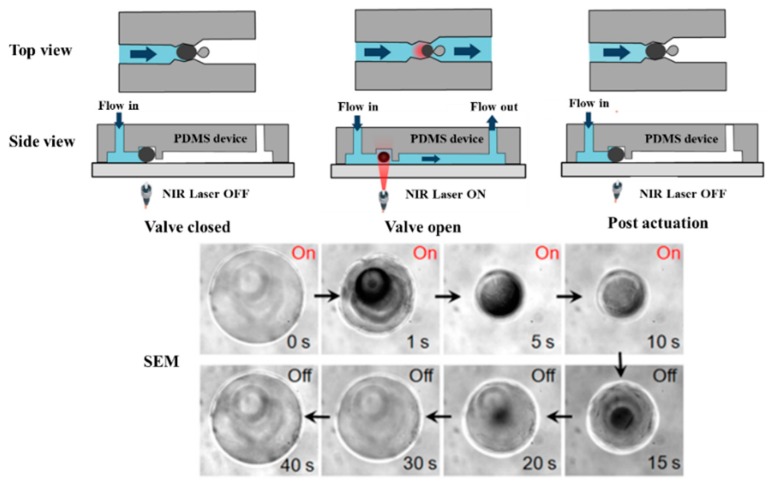
Schematic and SEM of photoresponsive hydrogel microvalve [97].

As mentioned above, the invisible lights including ultraviolet ray and infrared ray are also used to actuate the photoresponsive microvalves, and the light sources should choose some specialized light emitting devices. Jadhav et al. presented a novel photoresponsive hydrogel microvlave. The actuation principle was that the gel changed the volume by the near-infrared laser irradiation, and then the valve realized precise fluid on/off switching [97]. Figure 13 showed the operation of the valve and SEM of photoresponsive hydrogel. The irradiation power and time controlled the valve response speed and duration time. The proposed valve was suitable for fast fluidic switching applications.

**Figure 14:**
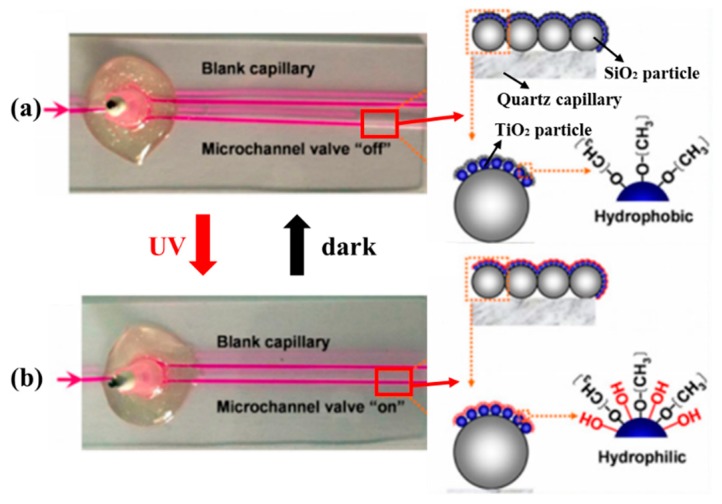
Schematic of UV/dark actuated wettability conversion in the surface of the microvalve [119]: (**a**) Off status and hydrophobic composite structure before UV irradiation; (**b**) On status and hydrophilic composite structure after UV irradiation.

Demir et al. presented a microvalve actuated by darkness and UV irradiation. The wettability conversion was used to adjust pressure drop in the capillary channels for switching two states (On/Off). The titanium microchannel was fabricated by the laser microdrilling, and chemical etching was applied to clear the remaining spatter for ensuring the quality of the hole [117,118]. The pressure drop could be increased by very small microchannel diameters and extreme values of the contact angle (i.e., 0° and 180°). Figure 14 showed schematic of UV/dark actuated wettability conversion in the TiO_2_/SiO_2_ composite surface of the microvalve [119]. The most important part of the valve was the novel micro-nano hierarchical structure, which could enhance the valve performances. The valve tests including reversible and repeated operations proved that the valve was good at adjusting microscale flow.

#### 2.4.2. pH-Sensitive Actuation

**Figure 15:**
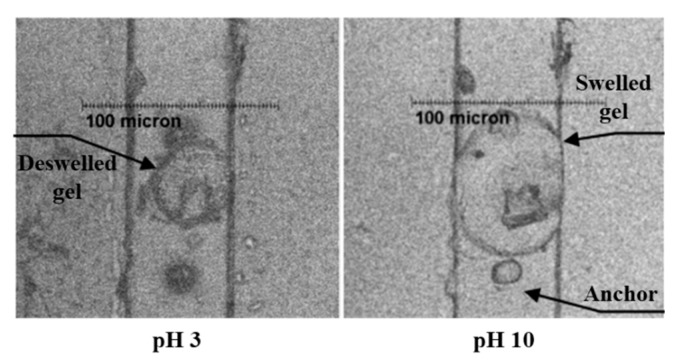
SEM of pH-sensitive microvalve [94].

As the soft material, hydrogels can undergo large deformation when they are stimulated by external force. The stimulation response of hydrogel to the change of pH value is used to fabricate the pH-sensitive microvalve. According to Figure 15, it is obvious that pH value is used to control microvalves by changing the volume of the pH-sensitive hydrogel. When the pH-sensitive hydrogel is in an alkaline environment, its volume will swell correspondingly. Arbabi et al. investigated a novel pH-sensitive microvalve based on a hydrogel jacket. The effects of different parameters, including inlet pressure, pH value and jacket patterns, were analyzed by fluid-structure interaction simulations, which were found to be important to accurate design of this microvalve [120]. Further, pH-responsive microvlaves could also be made into a valve-array for drug discovery [115]. The technology of photo-polymerization called “on the fly” was used to fabricate the device, and it had good performances through tests. The advantages of the valve array were obvious, like high-density distribution, no electrical modules, and so on.

#### 2.4.3. Glucose-Sensitive Actuation

The glucose-sensitive microvalve is suitable for the drug delivery system in human body, especially the patients with diabetes. A glucose-sensitive actuator applied to the microvalve was invented for the drug delivery system [116]. The microchannel is essential to carry liquid samples in the system, and two types of microchannel, rectangular and trapezoidal, were fabricated using anisotropic etching of deep-RIE and wet chemical etching, respectively. Through this microvalve, the insulin could be released cordially to human body automatically based on the glucose concentration. This hydrogel-actuated microvalve responded to the changes in the concentration of glucose in an external liquid environment.

#### 2.4.4. Paraffin Phase Transition Actuation

Because of the low melting point, paraffin wax can easily complete phase transition by heating. The microvalve exploits paraffin wax of low melting point, whose solid-liquid phase changes allow the closing and opening of fluid flow through deformable microchannel membrane. Valve switching is controlled by melting of paraffin through heating. The thin channel ceiling of the valve separates the fluid channel from the wax chamber, and the purpose is ensuring the fluid in the channel being free of contamination by the paraffin wax. This kind of microvalve needs a micro chamber to store the paraffin wax and a micro heater to heat it. The response times of opening and closing are relatively slow.

**Figure 16:**
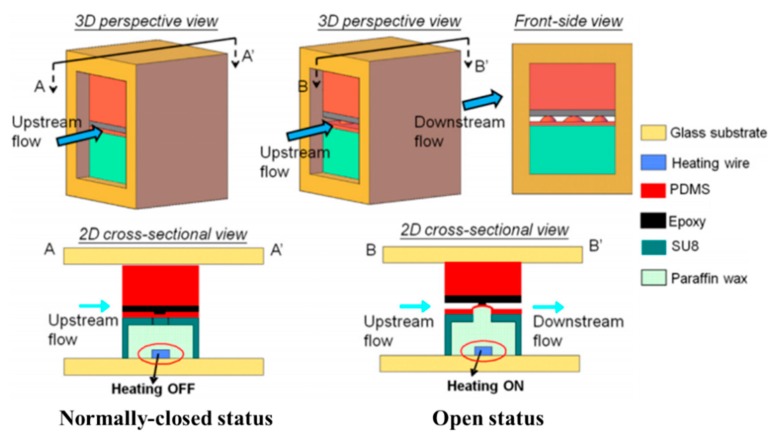
Working principle of the paraffin microvalve [102].

A silicon membrane optically driven restrictor microvalve was based on the paraffin mixed with optically absorbing nanographite particles [98]. The paraffin composite as an adhesive layer sandwiched between the silicon valve and the glass. The device was appropriate for high pressure and low volume flow applications. Yang et al. established a new latchable phase change microvalve utilizing paraffin wax. The channel of flow was controlled by solid-liquid phase changes of paraffin wax, and only valve switching process needed low power consumption for producing pneumatic pressure and heating [99]. Moreover, the proposed structure made the valve have good leakproofness, and isolated the flow channel from the chamber of wax by the channel ceiling. Although the switched state was maintained after paraffin solidified without further energy consumption, the response time of closing or opening was more than 60 s. The group of Yoo introduced a microfluidic system including a paraffin actuated microvalve and a thermopneumatic micropump for a micrototal analysis system and lab-on-a-chip. The material of the device contained PDMS-glass chip and an indium tin oxide (ITO) heater. This system realized accurate fluidic control, cost effectiveness, and portability [100,101]. A normally closed paraffin-actuated microvalve consisted of an elastic blockade and a fast heating microheater [102]. Micro-bulges were produced by the volume expansion of paraffin wax inside the microheater. As shown in Figure 16, the protruding micro-bulges produced by phase change of paraffin wax lifted the valve blockade, allowing fluid to pass. The valve was capable of realizing rapid switch-on, just 0.125 s, under a 3 V battery-powered supply. Baek et al. established a wireless microvalve system. Programmable opening of valves was designed by using different thermal responses of metal discs to a magnetic field. The discs as heating elements, controlled by induction heating, heated the flow plug (paraffin wax) to adjust fluid flow. The system was proved that it was inexpensive and easy to manipulate [103].

#### 2.4.5. Metal Phase Transition Actuation

##### Low Melting Point Alloy

**Figure 17:**
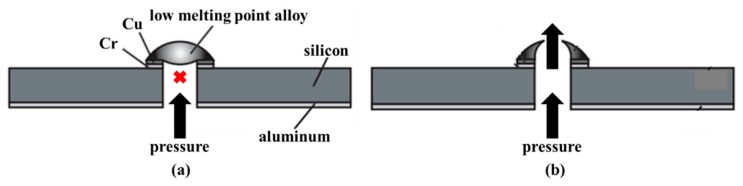
Scheme of the cross-section of the one shot microvalve based on low melting point alloy [105]: (**a**) In the closed position; (**b**) In the open position.

Low melting point alloy can be seen as the phase change material, and the melting temperature of it is relatively low. Some metal alloy pieces liquefy when they are heated above 62 °C. Indium-bismuth (In-Bi) and tin-lead (Sn-Pb) are typical low melting point alloys. A thin-film metal heater could be integrated into the device to provide localized heating. This kind of alloy is widely used in the one-shot microvalve, which means the valve is single use. Debray et al. developed a one-shot valve including a membrane coated with a low melting point alloy. The microvalve adopted the suspended metallic structure, and the manipulation of the valve depended on surroundings temperature and pressure [104,105]. As a normally closed microvalve, the valve opened when the ambient temperature was higher than the alloy melting temperature. What is more, the opening pressure difference across the channel was fairly low. The microvalve could be also opened if the pressure difference was such as to fracture the membrane in Figure 17. Manginell et al. focused on the materials about the phase-change microvalve for greenhouse gas (GHG) sampling. They proposed the low-melting-point eutectic metal alloys, which could be melted at 72 °C. As a low power, low weight, and low-cost alloy, this material decreased the leakage rate of the valve, and the device could be stored for a long time (2.8 years) [29]. Shaikh et al. presented a latchable microvalve using low melting point alloy to hold the valve in place when latched. Choosing metal alloy as the structural support instead of paraffin wax, etc, could guarantee the valve that had a higher burst pressure. The valve did not need extra power, and it was suitable for low-power portable lab-on-a-chip applications and long-term fluid storage [106].

##### Shape Memory Alloy (SMA)

Shape memory alloy (SMA), which consists of two or more metallic elements, has shape memory effect (SME). SMA is the best shape memory material at present. The main function of it is that the deformation of it at low temperature is eliminated by heating. Therefore, this kind of material has a wide range of applications in clinical medical field. SMA is widely used to fabricate microvalves. Ni-Ti shape memory alloy is used in some microvalves, and the shape memory alloy wire actuator provides robust flow control at a high pressure drop. The manufacture method of SMA is similar to the ordinary alloy, including melting, mechanical alloying, sintering, and vapor deposition. Further, SMA has many applications and methods of operation. Take a spring made of SMA as an example, when this spring is placed in hot water, the length of the spring is immediately extended, and then placed in cold water, it immediately returns to its original state. SMA is widely made into fire alarms and safety devices for electrical equipment, artificial bones, and so on.

**Figure 18:**
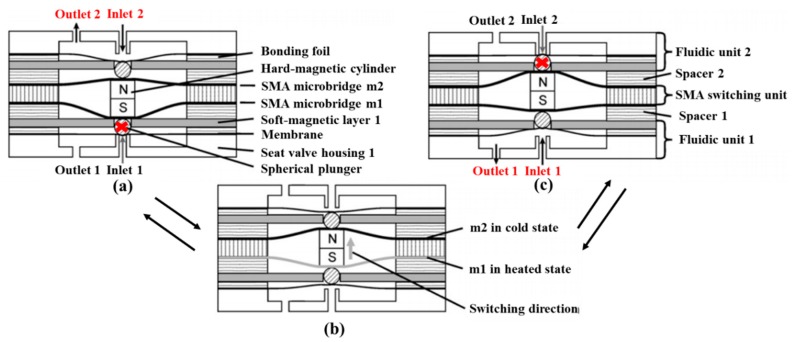
Schematic cross-section of the three-way bistable SMA microvalve [108]: (**a**) State I; (**b**) Switching state; (**c**) State II. (b) Switching from state I to state II is performed by directly heating microbridge m1 with an electrical current.

The group of Barth and Megnin presented a bistable SMA microvalve including two counteracting SMA microbridges and magnetic layers. Figure 18 displayed the schematic cross-section of the three-way bistable SMA microvalve under three states, including State I, State II and switching state. A modular layout and a novel self-aligning valve stack were important to fabricate the valve. Thus, the high requirement on vertical alignment accuracy (1 μm) was satisfied. Furthermore, another bisTable 3/2-way SMA microvalve realizing the bi-directional switching was proposed. The magnetic retaining system of the valve provided large adjustable pressure differences range and a low leakage rate. The valve could bore a pressure difference up to 200 kPa for gas (N_2_) and up to 100 kPa for liquid (water), respectively [107,108]. Gradin et al. investigated a SMA wire gas microvalve, which was suitable for high pressure high flow control [109]. Compared with the current high-flow valves in Table 7, the proposed design was good at the robust actuator performance, low power consumption and rapid response. The SMA wire actuator valve has more than one order of magnitude lower power consumption than the light actuated valve [97] and has two orders of magnitude lower voltage than the electrostatic microvalve [32] and piezoelectric microvalve [50]. The main drawback of the SMA wire valve compared to the other valves is the high relative leakage. However, this can be addressed by an improved design.

Two types of microvalves based on surface acoustic wave (SAW) were presented by Zhang et al. and Liu et al. They all comprised of the SMA wire and opening or closing of the valve was controlled by SAW, but their working principles are different. These two microvalves could be utilized in piezoelectric microfluidic devices for biochemical analysis [110,111]. Nath et al. researched a SMA microvalve based on laser actuation. As a contactless type, the laser was a low power density source, which was suitable to be chosen as actuation medium. The array of this type microvalve for flow control on macroscopic level was also presented. The flow flux about the microvalve array could be varied with opened valve number [112]. The comparison between low melting point alloy and SMA is displayed in Table 8. Based on the change of temperature, the state of the two alloys both also change: one undergoes the phase change and the other undergoes the deformation. Therefore, according to their respective characteristics, the former is mainly used for one-shot microvalves, and the latter is mainly used for the field of biomedicine.

#### 2.4.6. Biology Actuation

**Figure 19:**
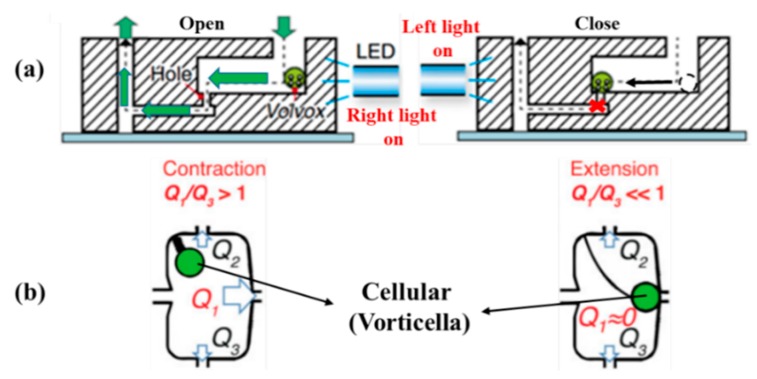
Schematic of two microvalves based on microorganism [113,114]: (**a**) V. carteri [113]; (**b**) Vorticella [114].

Microvalves based on biology actuation are rare in microfluidic systems. Some microorganisms and bacteria are used to act as the movable microvalve element assembled in microfluidic devices. According to Nagai et al., there is a type of a *Volvox* called *V. carteri* having phototaxisA novel light-controlled microvalve based on Volvox actuation was displayed in Figure 19a, and the PDMS structure of three layers with a through-hole was manufactured to satisfy the complex fabrication process. The phototactic behavior of *V. carteri* and controlled its motions in a microchannel by illuminating light. *V. carteri* migrated to the light source in the channel. Compared to the flow without Volvox on the hole, the colony of *V. carteri* was found to stop the flow. What is more, they also presented a Ca^2+^ driven bioactuator, which was the contractile fiber from the stalk of Vorticella cell. The valve chamber was used to grow the Vorticella cell. The behavior of stalk (contraction and extension) was controlled by the concentration of Ca^2+^, and then a cell body connected to the stalk opened or closed the micro channel. As shown in Figure 19b, vorticellas are selectively placed in chambers with channels. The vorticellas fuse with the artificial structure to develop a 2D actuator. Then permeabilization is performed to make the vorticellas controllable, solution containing Ca^2+^ is supplied, and reliable motion control is implemented according to the Ca^2+^ concentration in the solution. The microvalve based on this bioactuator was suitable for the integration and functionalization of microsystems [113,114].

Table 9 shows the comparison of four types of microvalves based on the properties of material and creature. Hydrogel, paraffin wax, and microorganism are used as driving sources in microvalves. These microvalves generally have lower energy consumption and the materials used are less polluting to the environment. The glucose-sensitive hydrogel, because of its high biocompatibility, is widely used for insulin injection in diabetic patients. Thus, these valves have great advantages for protecting the environment.

### 2.5. Surface Acoustic Wave (SAW)

**Figure 20:**
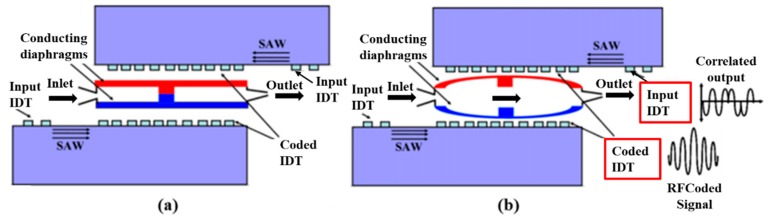
A SAW Microvalve in (**a**) the OFF/normally closed state; (**b**) the ON/open state [122].

Surface acoustic wave (SAW) is an elastic wave propagating along the surface of an object. The development of SAW technology has been greatly accelerated by the invention of a coded interdigital transducer (IDT), which responds only to a coded signal. The SAW device consists of an input IDT and a coded output IDT deposited on top of a piezoelectric substrate, which is shown in Figure 20 [121]. The input IDT transduces the coded input RF signal into an acoustic wave [122]. Figure 20 also displays the structure of the SAW based microvalve in the OFF/normally closed state and the ON state. In ON–OFF switching applications, the valve efficiency of an active microvalve with diffuser elements is poor in the reverse direction due to high leakage. Thus, the microcheck valve is installed in the fluid channel to realize the desired leak-tight operation. In the ON state, when interrogated by a correlating signal the double membranes inflate due to electrostatic actuation and inhale the fluid into the chamber [122].

Many microvalves based on SAW are utilized to for the purpose of secure wireless actuation. There are lots of advantages in the realization of a SAW microvalve including secure [122], reliable and low power operation [121], small size [123], simplicity in construction [124] and cost effectiveness [125]. Such microvalves have a huge range of applications such as in micro electro-mechanical systems (MEMS), nano electro-mechanical systems (NEMS) [121], biomedical applications, lab-on-chip applications [124], drug delivery [125,126], and so on.

The group of Dissanayake developed a novel wirelessly driven microvalve based on SAW. It did not require battery, and the safety of it was ensured by a coded SAW correlator. They also researched a microvalve utilizing radio frequency (RF) control on a PZT substrate for the application of biomedicine. For the wireless aspect of the whole device, the RF antenna was added in the SAW microvalve. A parallel type piezoelectric bimorph actuator was designed for achieving a better coupling between electrical signal and mechanical actuation [121,124].

According to Tikka et al., an SAW microvalve for long-range control was presented, including an electrostatic microchannel, two conducting diaphragms and two acoustic wave correlators. The safety of the valve was proved by FEM. SAW microvalve using the technology of inductively coupled RF, was studied for human body implant and long-distance drug delivery. The BPSK signal and the interrogator were the important part to achieve the contactless control of valve through near-field inductive coupling. The numerical model of it was established, and the results of simulation revealed that the device had advantages of small size and long working life. They also presented the method to improve the security of the wirelessly actuated microvalve. The valve was designed to be driven by the minimum value about electromagnetic (EM) radiation, considering existing EM radiation. Length about the required code was researched to guarantee safe manipulation [122,123,125,126]. Zhang et al. reported a novel SAW microvalve consisted of the piezoelectric substrate and an interdigital transducer (27.5 MHz). The working principle of the device was that the paraffin location was adjusted by the phase change of paraffin from solid to oil, to realize the valve manipulation. And red dye solution was applied to demonstrate the operation of the device and characterize the performance of it [127].

## 3. Future Research

The works that have considered microvalves are valuable, but by reviewing the literature, it is believed that further improvement and trial are still needed. Although the performance of microvalves have been improved in recent years, there are many disadvantages still existing, including high energy consumption, high cost, complex structure and the leakage of the whole microfluidic system. Because of the complex structure and many components, the traditional mechanical actuated microvalves cannot be completely integrated with the microfluidic system, resulting in the leakage problem. The active microvalves having externally driven devices, power consumption and portability still are big problems. The heat dissipation problem with external driven devices also effects the performances and accuracy of the microvalves. As the scope of application continues to expand, scientists have put forward the higher performance of microvalves. Current microvalves often only meet one certain requirement and they cannot meet multiple requirements at the same time. Therefore, in order to further improve the performances of microvalves, the research of microvalves can be started from the following aspects:

1. Lightweight material. Unlike traditional valves, the main feature of microvalves is their light weight. The use of lightweight materials can reduce the weight of the microvalve and improve its portability. The change from the metal material to polymer material is an obvious trend. However, the performance of the lightweight material should be improved. For example, when the microvalve is utilized in the outer space, the material should have a wider operating temperature range and work normally at low temperatures. It also has to withstand greater pressure differences. Anti-staining ability is also one of the most important characteristics of the material. New materials like nanomaterials could be used in microvalve manufacturing.

2. Integrated processing technology. Leakage is mainly caused by the improper assembly of components. The more components, the more assembly steps, the greater the leakage rate is. Integrated processing technology reduces the number of components in microvalves and the area of the dead zone, the accuracy of the fit is guaranteed to be below the micrometer. The development of micromachining is of benefit to integrated processing technology of the microvalve, like laser etching, rapid prototyping, and so on. Packaging is also a big problem in microvalves. Meanwhile, 3D printing technology is a choice that can be used for microvalve manufacturing.

3. Controlling performances. There are more and more actuation mechanisms applied to microvalves, and controlling performance depends on the actuation mechanism. Control accuracy and reaction time are two important indicators in the controlling performance of microvalves. Controlling fluid flow is one of the most important functions of microvalves, and the controlling performance of microvalves should be improved by optimizing actuation mechanism.

4. Flow characteristics. Due to the influence of microscale effects, the surface force cannot be ignored, so the flow of fluid in the microfluidic system is different from the macroscopic field [128]. It is necessary to establish a complete theoretical model of the microvalve. The numerical simulation method is combined with the experiment to study the internal flow mechanism of the microvalve, which is beneficial to the manufacture of microvalves, reduce costs and increase efficiency. What is more, common problems in valves like cavitation and vibration also compare in microvalves. Thus, the investigation of flow characteristics of fluids at the microscale is significant. The analysis of flow characteristics in industry valves is relatively complete, and is of great significance for studying the flow characteristics of microvalves.

5. Applications. Microvalve can save energy and provide precise control of the fluid. Up to now, little microvalves are utilized in the outer space. The microvalve is also a good choice to be used in the fuel cell, especially the hydrogen vehicles. Due to safety issues, hydrogen vehicles are highly demanding for hydrogen flow control, so microvalves may be a good alternative. In addition, the application of microvalves in the human body has become a trend [129], and microvalves have a good effect on eliminating effusion in certain organs. The biocompatibility of microvalve materials needs to be considered firstly.

## 4. Conclusions

With the rapid development of microfluidic technology, microvalves have received more and more attention from scientists. To improve the performance of microvalves, plenty of new structures and new materials are proposed to be utilized in the valve. New working principles have obviously reduced the cost, leakage rate, power loss, and dead zone of the microvalve, and increased the response speed and the biocompatibility. Microvalve applications are rapidly expanding from initial laboratory biochemical analysis to many other areas. More and more microvalves are applied in the human body to cure disease, like the brain, the eyes, and the blood vessel. Some microvalves are even utilized for the application of micro fuel cells. Many microvalves have many special actuation mechanisms based on their unique application environment, such as light actuated microvalves, biological actuated microvalves and glucose-sensitive actuated microvalves. Most early microvalves based on MEMS technology are mechanically actuated microvalves, while metal materials and silicon materials are utilized to fabricate microvalves by the multi-layer silicon process. Due to the complex structure of the device, it is difficult to integrate with the microfluidic system and it has many problems like high cost, poor reliability, high power consumption, and leakage problem. With the development of non-traditional manufacturing technologies, materials of microvalves have gradually transformed from silicon to polymer and PDMS is the most commonly used polymer material in microvalves. These microvalves are integrated easily with the microfluidic system. Low cost, good sealing performance, low leakage, and a small dead volume are the main advantages of these microvalves.

## Figures and Tables

**Table 1:** Piezoactuator specifications [41].

Stroke	Stiffness	Dimensions	Material	Unloaded Resonant Frequency	Block Force Estimate
80 µm	0.5 N·µm^−1^	8 × 10 × 17 mm^3^	Stainless steel amplification frame	1700 Hz	40 N

**Table 2:** Comparisons of three types of electrically actuated microvalves.

Type	Component	Advantage	Disadvantage	Application
Electrostatic	Electrodes; membrane	Low energy consumption; rapid response;	High applied voltage	high pressure gas control; chip; direct methanol fuel cell systems
Electrochemical	ECM; valve diaphragm	Precise adjustment	Complex structure; slow operation speed	Lab-on-a-chip; microfluidic system
Piezoelectric	Crystal, membrane	Large driving force; rapid response; high tolerance; low cost	High applied voltage;	Drug delivery system; micro-satellites

**Table 3:** Comparison of two types of magnetism driven microvalves.

Type	Components	Advantages	Disadvantages	Applications
Magnetic	Permanent magnet; elastic membrane with soft magnetic material	No energy consumption; simple structure; remote operation	Leakage	Microfluidic device; aeronautic flow control tests
Electromagnetic	Electromagnet	High precise control; rapid response	High energy consumption	Lab-on-a-chip

**Table 4:** Three design principles of pneumatic microvalves. The marking *LC* stands for liquid channel, *P* is for pressure, indicating the volume where the pressure for opening or closing is applied, cross-hatch is a PDMS layer, diagonal-hatch glass or thermoplastic layers [74].

No	Design Principle	Reference	Structure	Pressure (kPa)	
				Open	Close
1	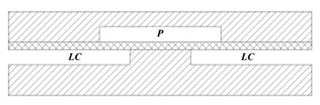	Perdigones, F. et al. [75]	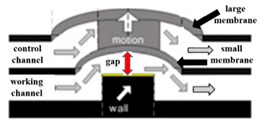	1	0
		Goldowsky, J. et al. [74]	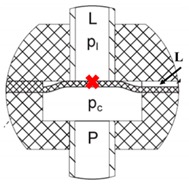	0	100
2	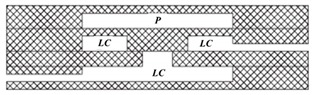	Baek, J.Y. et al. [76]	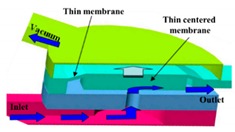	−2	40
3	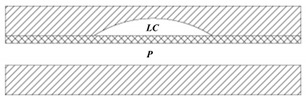	Samuel, R. et al. [77]	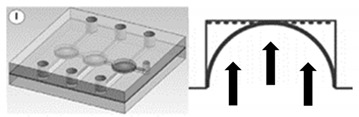	0	27.6 psi (4)

**Table 5:** Summary of microvalves based on pneumatic actuation and thermopneumatic actuation. P: Pneumatic; TP: Thermopneumatic.

Reference	Year	Type	Material	Medium	Fabrication	Application	Advantages
Shinichi et al. [72]	2015	P		Air flow		Wearable Robotic Systems	Light
Perdigones et al. [75]	2011	P	SU-8, gold	Gas	Post-exposure bake (PEB), BETTS process	Flow control, microfluidic circuits design	
Satoh et al. [78]	2008	P	Pyrex glass; silicon	Liquefied gases	Sandblasting, anodic bonding, long EPW etching	Liquefied gas control	High pressure leak tolerance, low pressure loss
Jamshaid et al. [79]	2013	P	PDMS, SU-8	The continuous flow (oil)	Standard soft lithographic technique	Droplet merging system	No desynchronization problem
Chen et al. [80]	2016	P	PDMS	Oil, water		Microfluidic droplets sorting	
Cong et al. [81]	2016	P	PDMS	hydrodynamic sample	Multilayer soft lithography	Microchip electrophoresis	Rapid analyte concentration, high sensitivity
Schneider et al. [82]	2015	P	a Si-C (silicon carbide) bottom electrode, aluminum amorphous silicon	Gas	Reactive Ion Etching (RIE), Deep Reactive Ion Etching (DRIE)	Reconfgurable tactile tablet for vision-impaired individuals	
Huang et al. [83]	2012	P	PDMS, PMMA		Hot embossing, irreversible bonding	Droplet generation, micro flow injection analysis	Reversible sealing
Kaminaga et al. [84]	2016	P	PDMS, SU-8, Si	The flow of the HeLa cell	Inclined lithography method	Conveying large cells	
Park et al. [85]	2012	P	PDMS, hardener, silicon wafers, gold microelectrode		Peel, punch, spincoat	Electrochemical microfluidic devices	
Perdigones et al. [87]	2014	TP	Flame Retardant 4, copper, gold wire, SU-8, PDMS	Fluid	Post exposure bake (PEB), the wet etching	Portable SU-8 Microfluidic Platforms	Independence of external pressure sources, high integrability, low consumption
Huesgen et al. [88]	2010	TP	Silicon	Fluid flow	Silicon technology	Liquid flow control	Low leakage rate, low energy cost
Mongprane et al. [89]	2009	TP	PDMS, microheater (NiCr), glass	Gas	PDMS spinning, oxygen plasma bonding, electroplated micromasking, thermal evaporation	Microfluidic Chip	Low cost fabrication
Aravind et al. [90]	2013	TP	Phase change liquid, PDMS, glass, silicon	Methanol, Isopropanol	Soft lithography, polymer processing	µTAS or Lab-on-chip	Precise control and manipulate liquid
Potkay et al. [91]	2012	TP	Glass, Si-glass, Au-Si	Fluid	Deep boron etch-stop, shallow isotropic etch, NaOH electrochemical etch, ethylene diamine pyrocatechol (EDP) etch	Electrostatic latching	Low energy consumption
Yang et al. [92]	2010	TP	Polymer, adhesive strip, ring magnet (Nd-Fe-B), silicone	Gas flow	Sputtering, photolithography		No leakage, no extra energy supply

**Table 6:** Microvalves based on material and biology properties. NC, normally closed microvalve; NO, normally open microvalve; B, bistable; SMA, shape memory arroy.

Reference	Mode	Type	Reversible/Irreversible	Phase Change Material/Creature	Max Pressure (no Leakage)	Time	Application
Al-Aribe et al. [93,94]	NO	Light	Reversible	HEMA-AA hydrogel (pH sensitive)			
Benito-Lopez et al. [95]	NC	Light	Reversible	Ionic liquid polymer gels (ionogels)		Open: seconds; Close: minutes	Microfluidic manifolds (single-use device)
Chen et al. [96]	NC	Light	Reversible	PNIPAM gel	1350 psi	Open: 4 s; Close: 6.2 s	Manipulate flow path in micro-total analysis systems
Jadhav et al. [97]	NC	Light (a near-infrared (NIR) laser)	Reversible	Microgel particles (PNIPAM)		Open: 1~2 s; Close: 6~8 s	Liquid handling in microfluidic devices
Kolari et al. [98]	NC	Paraffin	Reversible	Paraffin wax mixed with a suitable concentration of optically absorbing nanographite particles	2 bar		High pressure, low volume flow silicon-based nanofluidic systems
Yang et al. [99]	NO	Paraffin	Reversible	Paraffin wax of low melting point	35 kPa	Open: 100 s; Close: 60 s	Flow gating in portable lab-on-a-chip systems
Yoo et al. [100,101]	NO	Paraffin	Reversible	Thermally triggered phase change of the paraffin			Transport of reagents and samples for a lab-on-a-chip
Feng et al. [102]	NC	Paraffin	Reversible	Paraffin wax	25 kPa (backpressure)	Open: 0.125 s Close: 3.5 s	
Baek et al. [103]	NC	Paraffin	Reversible	Paraffin wax	107 kPa	Open: 1~5 s (short intervals); 15-23 s (long intervals)	Wireless sequentially actuated microvalve system
Debray et al. [104,105]	NC	Low melting point alloy	Irreversible	Alloy (Bi 44.7%, Pb 22.6%, In 19.1%, Sn 8.3%, Cd 5.3%) with a melting temperature of 47 °C	200 kPa	Open: 33 s	One-shot micro-valve
Shaikh et al. [106]	NO	Low melting point alloy	Reversible	A fusible metal alloy (Galinstan: 68.5% Ga, 21.5% In, 10% Sn) that is liquid at room temperature	138 kPa	Open: 100 ms up to 1 s	Portable lab-on-a-chip devices (low-power operation, long-term fluid storage)
Barth and Megnin et al. [107,108]	B	SMA	Reversible	SMA (A cold-rolled Ti-49 at.%Ni foil)	Gas (N2): 200 kPa Water: 100 kPa	200 ms	
Gradin et al. [109]	NC	SMA	Reversible	NiTi SMA wires	200 kPa	50 ms	High gas flow control
Zhang et al. [110], Liu et al. [111]	NO	SMA	Reversible	SMA wire	35 kPa	46 s (switch)	Piezoelectric microfluidic devices for biochemical analysis
Nath et al. [112]	NC	SMA	Reversible	NiTi SMA	5 kPa		Micro-valve array
Nagai et al. [113,114]	NO	Creature	Reversible	V. carteri (light-controlled Volvox)	50 mmH_2_O	30 s	Multilayer microfluidic device
	NC	Creature	Reversible	Cells of Vorticella convallaria		Contraction: 10.5 ± 3.57 s Extension: 24.4 ± 9.93 s	Compact and multifunctional microsystems
Liu et al. [115]	NC	pH (integrative micro-valve array)	Reversible	pH-responsive microspheres	50 kPa	Open: 60 s Close: 50 s	Drug discovery, high-throughput screening
Dzulkefli et al. [116]	NC	Glucose	Reversible	Glucose hydrogel			Drug delivery system (DDS)
Demir et al. [117,118]	NC	Light (darkness and ultraviolet (UV))	Reversible	TiO_2_ layers (wettability conversion)	980 Pa		
Guo et al. [119]	NC	Light (darkness and ultraviolet (UV))	Reversible	A trimethyl chlorosilane (CTMS) modified TiO_2_/SiO_2_		Minutes	Microscale flow control

**Table 7:** Comparison of typical high flow microvalves.

Reference	Type	Medium	Leakage (Relative or Absolute)	Power Consumption	Voltage	Response Time
Bae et al. [31]	ES	Gas	0		140 V	50 μs
Dankovic et al. [33]	ES	Gas	7.14% (max)		350 V	
Fazal et al. [41,46]	PE	Gas	0	low	2.5 V	
Park et al. [50]	PE	Gas	0	0.16 µW	60 V	0.7 ms
Wiederkehr et al. [55]	PE	Gas			300 V~−200 V	
Huesgen et al. [88]	TP	Liquid	1 μL/min	1 J (close); 2 J (open)		Close: 80 ms to 160 ms; Open: 240 ms to 400 ms
Jadhav et al. [97]	Light	Liquid	0	2.5 W		Open: 1–2 s; Close: 6–8 s
Guo et al. [119]	Light	Liquid	0			Minutes
Megnin et al. [108]	SMA	Gas/Liquid	<10 μL/min	60 mW		20 ms
Gradin et al. [109]	SMA	Gas	50%–70% (<10% possible)	90 mW	0.6 V	50 ms

ES, electrostatic; PE, piezoelectric; TP, thermopneumatic; SMA, shape memory alloy.

**Table 8:** Comparison of different metal materials applied to microvalves.

Type	Materials	Characteristics	Typical Application
Low melting point alloy	In-Bi; Sn-Pb	T > 62 °C: liquidation	One-shot microvalve (single use); greenhouse gas (GHG) sampling
SMA	Ni-Ti	Low temperature: deformation; High temperature: recover	Clinical medical field; high pressure high flow control; biochemical analysis

**Table 9:** Comparison of different microvalves based on properties of material and creature.

Type	Advantages	Disadvantages
Light	Long distance control	Long response time
pH	No energy consumption	Long response time
Glucose	High biocompatibility	Difficult to produce
Paraffin	Low cost	High energy consumption
Low melting point alloy	Reusable, easy to manufacture	High energy consumption
SMA	Shape memory effect	High energy consumption
Biology	No pollution	Long response time
